# Novel Drugs and Combination Therapies for the Treatment of Metastatic Melanoma

**DOI:** 10.14740/jocmr2424w

**Published:** 2015-12-28

**Authors:** Adarsh Vennepureddy, Nishitha Thumallapally, Vijeyaluxmy Motilal Nehru, Jean-Paul Atallah, Terenig Terjanian

**Affiliations:** a Department of Medicine, Staten Island University Hospital, Staten Island, NY 10305, USA.; b Division of Hematolgy and Oncology, Staten Island University Hospital, Staten Island, NY 10305, USA

**Keywords:** Metastatic melanoma, Targeted therapy, Immunotherapy, Combination therapy

## Abstract

Metastatic melanoma (MM) still remains as one of the most worrisome cancer known to mankind. In last two decades, treatment of melanoma took a dramatic turn with the discovery of targeted therapy which targets the mutations in mitogen-activated protein kinase (MAPK) pathway and immune checkpoint inhibitors. These new findings have led to emergence of many novel drugs that have been approved by FDA. Targeted therapy drugs such as vemurafenib, trametinib and dabrafenib target the MAPK pathway whereas immunotherapies such as ipilimumab, nivolumab and pembrolizumab block immune checkpoint receptors on T lymphocytes. All these drugs have shown to improve the overall survival in MM. Despite these recent discoveries, treatment of MM remains challenging because of rapid development of resistance to targeted therapy. This review will discuss recently approved drugs and their adverse effects and also shed light on combination therapy in treatment of melanoma.

## Introduction

Melanoma is the leading cause of death from skin disease. It has been reported as fifth and seventh most common cancer in USA in men and women respectively [1]. According to the National Cancer Institute (NCI), an estimated 73,870 new cases of melanoma will be diagnosed in the United States in 2015, and about 9,180 people would have died from the disease in 2014. The incidence of melanoma additionally varies by ethnic group. It accounts for 1 (per 100,000) in black people, four in Hispanics, and 25 in non-Hispanic whites annually [1]. The 10-year overall survival (OS) rate for advanced melanoma is about 10-15% and in the elderly (age > 70), regardless of their disease stage, the survival rate drops dramatically [2]. Majority of melanoma lesions are diagnosed early and are mostly excised and curable. But the real challenge lies in treating advanced melanoma. Treatment of melanoma depends on the stage on presentation. As per NCI, excision is treatment of choice for stage 0 melanoma. Stage II, III and resectable melanoma are managed with excision and lymph node resection if involved and unresectable stage III and IV melanoma are treated with help of chemotherapy, targeted therapy and immunotherapy [3].

Treatment of metastatic melanoma (MM) has changed drastically over the last decade. Historically, melanomas were considered as a single disease entity and treatment options included radiation therapy/surgery or chemotherapy with dacarbazine, an alkylating agent. However response to chemotherapy was nominal and durable remission rarely occurred. Encountered with un-satisfactory results with traditional chemotherapy, focus was shifted on learning in depth pathogenesis of melanoma at cellular and molecular level. With extended knowledge in molecular medicine, melanoma has been reclassified as a highly complex heterogenous disease comprising of several subpopulation of tumor cells. Number of gene mutations and aberrant cell signaling pathways have been recognized which led to development of targeted therapy and immunotherapy drugs. Although these new drugs show dramatic increase in overall response rate and extended survival, treatment of advanced melanoma still remains a challenge [4-6].

In recent years, four different classes of novel drugs were approved revolutionizing the care of advanced melanoma. These include immunotherapy (anti-cytotoxic T-lymphocyte antigen-4 (CTLA-4) monoclonal antibodies; anti-programmed cell death-1 protein (PD-1) monoclonal antibodies), targeted therapy like BRAF inhibitors and MEK inhibitors.

In this review, we discussed the development and current status of the expanding landscape of melanoma treatment.

## Mechanism of Action of Immunotherapy

Under normal physiologic conditions, the immune checkpoints serve to restrain immune responses against self-antigens, thereby preventing unwanted autoimmunity. However, these inhibitory pathways are up-regulated in many cancers, and immune checkpoints play critical roles in cancer-associated immune suppression and immune evasion [7].

### Anti-CTLA-4 antibodies

The primary effector cells of the adaptive immune response against cancer are the T lymphocytes which include both T helper cells and cytotoxic T lymphocytes. Cytotoxic T lymphocytes have direct tumor killing ability and T helper cells play a pivotal role in propagating anti-tumor response. T-cell activation requires two sequential signals. In a first step, antigens presented in context with the major histocompatibility complex (MHC) I or II on specialized antigen-presenting cells (APCs) bind with T-cell receptors (TCRs). The second step involves translation of TCR stimulation into T-cell activation and requires a co-stimulatory signal, achieved when B7 molecules on the APC surface bind with CD28 receptors on the T-cell surface. Subsequently, T-cell surface expression of an inhibitory molecule, CTLA-4, takes place. CTLA-4 competitively inhibits the binding of B7 to CD28 by interacting with the same ligands and prevents the co-stimulatory signal, dampening T-cell activation and proliferation. CTLA-4 thereby serves as a physiologic “brake” on the activated immune system [8-11].

### Anti-PD-1 antibodies

A second co-inhibitory pathway uses the PD-1 receptor, which is another inhibitory receptor present on activated T cells. PD-1 is a protein that is encoded in humans by PDCD gene. PD-1 is a cell surface immune checkpoint receptor which belongs to the immunoglobulin super family and is expressed on T cells and pro-B cells. PD-1 binds to its two ligands, PD-L1 and PD-L2 which are the members of B7 family. PD-1 and its ligands play an important role in down regulating the immune system by preventing the activation of T cells, which in turn reduces autoimmunity and promotes self-tolerance. The inhibitory effect of PD-1 is accomplished through a dual mechanism of promoting apoptosis (programmed cell death) in antigen specific T cells in lymph nodes while simultaneously reducing apoptosis in regulatory T cells (suppressor T cells).

PD-1 binds to its ligands PD1-L1 (B7-H1) and PD1-L2 (B7-DC), which are expressed on tumor cells, thereby causing immunosuppression and preventing the immune system from rejecting the tumor. When PD-1 binds to its ligand (PD-L1) (often present on tumor cells), the ability of the activated T cell to produce an effective immune response is down-modulated. Monoclonal antibodies targeting both PD-1 and PD-L1 are being developed to interrupt this pathway and to augment the antitumor immune response; these have demonstrated significant clinical activity against several tumor types [12, 13].

## Ipilimumab

Ipilimumab, an anti-CTLA-4 monoclonal antibody, is the first agent ever proven to improve survival in advanced melanoma. It was approved by Food and Drug Administration (FDA) in 2011. The current FDA approved dosing schedule for ipilimumab is 3 mg/kg intravenous (IV) infusion every 3 weeks (Q3W) for a total of four doses.

### Efficacy

Ipilimumab prolongs the survival rate in patient with MM and this has been confirmed in two large randomized phase III trials.

The first study by Hodi et al included 676 patients with unresectable stage III or IV melanoma whose disease had progressed after previous treatment. The patients were randomized in a 3:1:1 ratio to receive ipilimumab with the melanoma peptide vaccine gp100 (n = 403), ipilimumab with gp100 placebo (n = 137), or gp100 vaccine with ipilimumab placebo (n = 136). Ipilimumab was dosed at 3 mg/kg IV Q3W for a total of four treatments with the vaccine administered immediately after each ipilimumab infusion. The median OS at 20 months was 10, 10.1, or 6.4 months for patients treated with the combination, ipilimumab alone, or gp100 alone, respectively. The increased survival in both ipilimumab containing regimens was statistically significant. One- and 2-year OS rates for ipilimumab alone were 45.6% and 23.5%, respectively; for gp100 alone, 25.3% and 13.7%, respectively; and for ipilimumab plus gp100, 43.6% and 21.6%, respectively [14].

In a second phase III study which was conducted by Robert et al, 502 treatment-naive patients with unresectable stage III or IV melanoma were randomly assigned to in 1:1 ratio to receive either dacarbazine with ipilimumab (n = 250) or dacarbazine with placebo (n = 252) given at weeks 1, 4, 7 , 10. Ipilimumab was administered at a higher dose of 10 mg/kg for four doses followed by a maintenance phase. The addition of ipilimumab to dacarbazine significantly improved the primary outcome of OS compared with dacarbazine alone (11.2 vs. 9.1 months; P < 0.05) [15].

A recent study published on February 9, 2015 was a pooled analysis of long-term survival data from phase II and III trials of ipilimumab in unresectable MM. The main purpose of the study was to give a precise estimate of long-term survival for ipilimumab-treated patients. The study included pooled OS data for 1,861 patients from 10 prospective and two retrospective studies of ipilimumab, including two phase III trials. Among 1,861 patients, median OS was 11.4 months (95% CI, 10.7 - 12.1 months), which included 254 patients with at least 3 years of survival follow-up. The survival curve began to plateau around year 3, with follow-up of up to 10 years. Three-year survival rates were 22%, 26%, and 20% for all patients, treatment-naive patients, and previously treated patients, respectively. These data once again support the durability of long-term survival in ipilimumab-treated patients with advanced melanoma [16].

### Usage in other cancers

Ipilimumab is under various phases of clinical trials for the treatment of metastatic renal cell [17], prostate [18] and non-small cell lung cancers [19].

## Pembrolizumab

Pembrolizumab is an anti-PD-1 monoloclonal antibody that has been extensively evaluated in ipilimumab-naive and previously treated melanoma patients. Pembrolizumab at a dose of 2 mg/kg IV Q3W was approved by the US FDA in September 2014 for patients who have progressed on treatment with ipilimumab.

### Efficacy

Pembrolizumab was awarded FDA approval based on the following data from an international multicenter, open-label, randomized, dose-comparative phase 1 study randomizing 655 patients (342 ipilimumab treated (IPI-T) and 313 ipilimumab naive (IPI-N)) with unresectable or MM to receive pembrolizumab 2 mg/kg or 10 mg/kg IV once Q3W or 10 mg/kg every 2 weeks (Q2W). In the published analysis of 173 patients from this trial with ipilimumab-resistant disease who were assigned to either 2 mg/kg or 10 mg/kg Q3W, objective response rate (ORR) was achieved in 26% in both treatment arms with response duration lasting from 1.4 to 8.5 months (KEYNOTE-001 trial) [20].

The results of all the patients enrolled in this trial were recently published at American Society of Clinical Oncology (ASCO) 2015 annual meeting. ORR was 34% (29% IPI-T, 38% IPI-N), with a 6% complete remission (CR) rate. Median time to response was 2.8 months (range, 1.6 - 19.3). Eighty percent of responses were ongoing at the time of analysis, and median duration of response (DOR) was not reached (range, 6+ to 98+ weeks). Median progression-free survival (PFS) was 5.2 months (IPI-T, 4.9 months (3.0 - 5.5); IPI-N, 5.4 months (3.1 - 6.9)). PFS rates at 6 and 12 months were 44% and 34% (41% and 32% IPI-T, 47% and 36% IPI-N). The 1-year OS rate was 67%. Pembrolizumab provided durable antitumor activity, promising long-term survival data, and a manageable safety profile in both ipilimumab-naive and treated patients with MM [21].

In the KEYNOTE-002 trial which was a phase II trial, 540 patients with ipilimumab refractory MM were randomly assigned to pembrolizumab (2 mg/kg Q3W), pembrolizumab (10 mg/kg Q3W) or chemotherapy (carboplatin plus paclitaxel, paclitaxel alone, dacarbazine, or temozolomide per institutional standard) [22]. Treatment continued on this schedule until progressive disease. The ORRs (complete plus partial) were 21%, 26%, and 4% respectively, for pembrolizumab 2 mg/kg, pembrolizumab 10 mg/kg, and chemotherapy. Treatment was relatively well tolerated, with grade 3-5 adverse events (AEs) reported in 11% and 14% of the pembrolizumab treatment arms, and 26% of those managed with chemotherapy.

The ability of ipilimumab-refractory disease to respond to pembrolizumab is probably a reflection of the different mechanisms by which anti-CTLA-4 and anti-PD-1 therapies stimulate an anti-tumor T-cell response. CTLA-4 blockade broadens the immune response, evidenced by an increased T-cell receptor repertoire leading to increased tumor infiltration, whereas PD-1 blockade induces intratumoral T-cell proliferation without detectable changes in the peripheral immune repertoire.

Robert et al (KEYNOTE-006) have recently finished a randomized phase III trial of pembrolizumab in patients with advanced melanoma. Eight hundred thirty-four patients were assigned in a 1:1:1 ratio to receive pembrolizumab (at a dose of 10 mg/kg) Q2W or Q3W or four doses of ipilimumab (at 3 mg/kg) Q3W. The 6-month PFS rates were 47.3% for pembrolizumab Q2W, 46.4% for pembrolizumab Q3W, and 26.5% for ipilimumab. Estimated 12-month survival rates were 74.1%, 68.4%, and 58.2%, respectively. Treatment experienced adverse events (TEAEs) of grade 3-5 severity were lower in the pembrolizumab groups (13.3% and 10.1%) than in the ipilimumab group (19.9%) [23].

### Usage in other cancers

Pembrolizumab has demonstrated highly durable response rates with minimal toxicity in large phase I studies involving patients with non-small-cell lung cancer (NSCLC), renal cell carcinoma, and other solid tumors [20, 24]. It has been FDA approved recently for the treatment of advanced NSCLC as a second-line treatment [25].

## Nivolumab

Nivolumab is a monoclonal antibody that targets the PD-1 protein. Nivolumab at a dose of 3 mg/kg IV infused over 60 min Q2W was approved by US FDA in December 2014 for patients who progressed after treatment with ipilimumab and in patients whose tumors express BRAF V600 mutation.

### Efficacy

In a phase I trial designed by Topalian et al, 107 patients were treated with nivolumab at doses from 0.1 to 10 mg/kg Q2W for up to 96 weeks. The results of this study showed a median survival of 17 months and CR or partial remission (PR) rates were observed in 34 of 107 patients (32%). One-year and 2-year survival rates were 62% and 43% respectively [26]. Expression of PD-L1 by the tumor appeared to predict for a higher response rate, long-term PFS, and long-term OS compared with tumors that did not express PD-L1. These results led to the conduction of further clinical trials.

In previously untreated patients, Robert et al conducted a phase III trial (Checkmate 066) (NCT01721772) [27]. Four hundred eighteen previously untreated MM patients without BRAF mutation were randomly assigned to nivolumab (at a dose of 3 mg/kg Q2W and dacarbazine-matched placebo Q3W) or dacarbazine (at a dose of 1000 mg/m^2^ of body-surface area Q3W and nivolumab-matched placebo Q2W). OS was significantly increased in those treated with nivolumab (1-year survival rate 73% versus 42%). PFS was also increased with nivolumab (median 5.1 versus 2.2 months), as was the ORR (40% versus 14%). Common AEs associated with nivolumab included fatigue, pruritus, and nausea. TEAEs of grade 3 or 4 occurred in 11.7% of the patients treated with nivolumab and 17.6% of those treated with dacarbazine.

In previously treated patients, Weber et al performed a randomized, controlled, open-label phase III trial to assess the efficacy and safety of nivolumab compared with chemotherapy as a second-line or later-line treatment in patients with MM (Checkpoint 037) (NCT01721746). All patients had received prior anti-CTLA-4 therapy and a BRAF inhibitor if a V600 mutation was present in their tumor. Two hundred seventy-two patients were randomly assigned to nivolumab group (3 mg/kg IV infusion Q2W) and 133 patients were assigned to chemotherapy group (either dacarbazine or carboplatin plus paclitaxel). The trial accrued 405 patients; preliminary results based upon 167 patients (120 treated with nivolumab and 47 with chemotherapy) showed that confirmed objective responses were significantly more common in patients treated with nivolumab compared with chemotherapy (32% versus 10%) [28]. Nivolumab led to a greater proportion of patients achieving an ORR and fewer toxic effects than with alternative available chemotherapy regimens for patients with advanced melanoma that has progressed after ipilimumab or ipilimumab and a BRAF inhibitor.

### Usage in other cancers

Nivolumab has been studied to show some clinical activity in the treatment of Hodgkin’s lymphoma [29, 30], in patients with advanced, refractory NSCLC [31] and in patients with metastatic renal cell cancer [32]. Nivolumab is also being investigated under phase I/II study as a monotherapy or in combination with ipilimumab in locally advanced or metastatic tumors including triple negative breast, small cell lung, gastric and pancreatic malignancies (NCT01928394). Similarly, a phase IIb randomized study is evaluating the efficacy and safety of nivolumab alone or with ipilimumab when compared to bevacizumab in patients with recurrent glioblastoma (NCT02017717) [33]. Nivolumab has been FDA approved recently for the treatment of both squamous and non-squamous advanced, refractory NSCLC as a second-line treatment [34, 35].

## AEs of Immunotherapy

Although the evolution of immune therapy antibodies can be associated with substantial benefits, by increasing immune system function, immune-checkpoint blockade can lead to inflammatory side effects called immune-related adverse events (IrAEs). IrAEs can affect any organ system, but they typically involve the skin, gastrointestinal, hepatic, and endocrine systems [36].

The most common IrAEs for both CTLA-4 and PD-1 therapy involve dermatologic toxicity. Physical examination findings can consist of a reticular, maculopapular, erythematous rash on the extremities or trunk [37]. Perhaps more unique to the PD-1 experience, oral mucositis and/or complaints of dry mouth have been reported in a small percentage of patients [26]. Fatigue is the most common side effect in patients with anti-PD-1 therapy.

Endocrinopathies affect the pituitary, thyroid, adrenal glands and manifest with non-specific symptoms such as nausea, headache, fatigue and vision changes. Diagnosis is usually made by characteristic laboratory findings and/or radiographic changes, such as enlargement of the pituitary gland [38].

Table 1 depicts the most common AEs and their grades.

**Table 1:** Adverse Effects of Immunotherapy and Management

Treatment related adverse effects	Grade 1	Grade 2	Grade 3	Grade 4
Skin toxicity. Most common adverse effect.	Mild to moderate localized rash or pruritus; papules/pustules covering < 10-30% of body surface. Rx: topical corticosteroids.	Non-localized rash (diffuse, ≤ 50% of skin surface) Rx: topical corticosteroids and monitoring.	Intense or widespread rash > 30%; skin sloughing < 10-30% of body surface; epidermal or mucus membrane detachment. Rx: systemic corticosteroids, hospitalization and hold immunotherapy.	Stevens-Johnson syndrome, toxic epidermal necrolysis (1% of cases), or rash complicated by full-thickness dermal ulceration, bullous and blisters. Rx: immediate hospitalization, systemic steroids and discontinue drug permanently.
GI toxicity/diarrhea. Second most common	< 4 stools per day over baseline Rx: symptomatic treatment.	4 - 6 stools per day over baseline. Rx: IV fluids for < 24 h, and symptomatic treatment. Rule out infectious causes. If not improving, hold drug and consider oral/IV steroids.	≥ 7 stools per day over baseline. Rx: IV fluids for > 24 h, hospitalization and IV steroids.	Life-threatening consequences (e.g., hemodynamic collapse). Rx: hospitalization, IV fluids, IV steroids. If symptoms not improving with IV steroids, consider infliximab.
Hepatotoxicity. Occurs in about 10% of patients.	Asymptomatic or mild symptoms. Rx: clinical or diagnostic observations only; intervention not indicated	AST or ALT > 2.5 to ≤ 5.0× ULN and/or total bilirubin > 1.5 to ≤ 3.0× ULN. Rx: frequent monitoring of LFTs. Consider holding immunotherapy.	AST or ALT > 5× ULN and/or total bilirubin > 3.0× ULN. Rx: hold immunotherapy and frequent monitoring of LFTs. Rule out viral, autoimmune or drug induced hepatitis.	High ammonia levels and hepatic encephalopathy. Rx: discontinue drug permanently and start high dose steroids (2 mg/kg/day). If not improving in 48 h, consider oral mycophenolate (500 mg twice daily).
Endocrine toxicity. Occurs in 4-8% of patients.	Asymptomatic. Rx: clinical or diagnostic observations only; intervention not indicated	Moderate symptoms. Rx: if suspicious for hypophysitis is high, complete endocrine workup should be done. Hormone replacements may be considered.	Severe symptoms. Rx: hospitalization indicated. Stop immunotherapy. Short course of steroids may improve pituitary function.	Adrenal crisis: severe dehydration, hypotension, or shock. Life-threatening consequences Rx: high dose IV steroids.

AST: aspartate aminotransferase; ALT: alanine aminotransferase; ULT: upper limit of normal.

## Management of AEs

For patients with moderate (grade 2) immune-mediated toxicities, treatment should be withheld and should not be resumed until symptoms resolve. Corticosteroids (prednisone 0.5 mg/kg/day or equivalent) should be started if symptoms do not resolve in a week. For patients with severe or life-threatening AEs, treatment should be stopped permanently and high-dose corticosteroids (prednisone 1 - 2 mg/kg/day or equivalent) should be given.

Patients who benefit from steroids generally do so within a few days. If symptoms do not improve after 3 days of treatment with IV steroids, next step is to administer infliximab (5 mg/kg) rather than continuing prolonged course of high-dose IV corticosteroids. In cases of severe hepatotoxicity, mycophenolate mofetil (500 mg orally every 12 h) can be administered concurrently with steroids and infliximab is contraindicated in such patients [39].

 IrAEs associated with CTLA-4 blockade increase with increasing dose, whereas IrAEs associated with PD-1 blockade do not appear to be dose-related. Some IrAEs are also associated with the targeting of one pathway but not the other [26].

## Mechanism of Action of Targeted Therapy

### BRAF and MEK inhibitors

BRAF is a human gene that makes a protein called B-Raf. The B-Raf protein is involved in sending signals inside cells, which are involved in directing cell growth. In 2002, it was shown to be faulty (mutated) in some human cancers. B-Raf is a member of the Raf kinase family of growth signal transduction protein kinases. This protein plays a role in regulating the mitogen-activated protein kinase (MAPK)/extracellular signal-regulated kinases (ERKs) signaling pathway, which affects cell division, differentiation, and secretion.

Mutations in BRAF gene can occur in two ways. It can be either inherited and cause birth defects or it can be acquired later in life and causes cancer. The frequency of BRAF mutations varies widely in human cancers, from more than 80% in melanomas and nevi, to as little as 0-18% in other tumors, such as 1-3% in lung cancers and 5% in colorectal cancer. In 90% of cases, thymine is substituted with adenine at nucleotide 1799 which leads to valine (V) being substituted for by glutamate (E) at codon 600 referred to as V600E mutation that activates the MAPK pathway. The V600K mutation results in an amino acid substitution at codon 600 in BRAF, from a valine (V) to lysine (K). This mutation has been widely associated with papillary thyroid cancer, NSCLC, colorectal cancer and melanoma.

**Figure 1:**
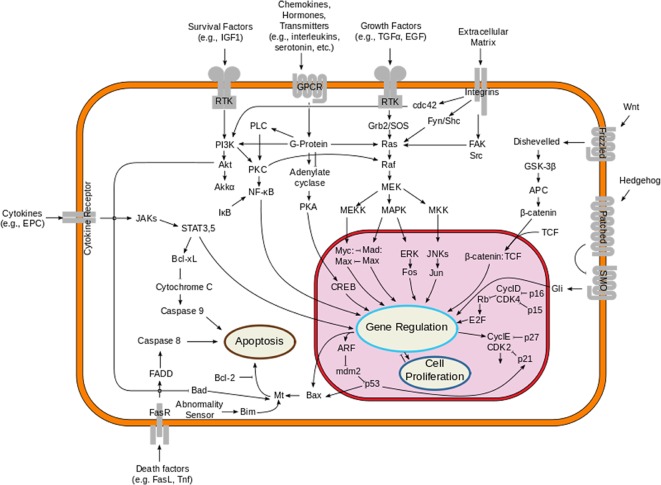
Overview of the signal transduction pathway like mitogen-activated protein kinase/extracellular signal-regulated kinases signaling pathway.

The MAPK pathway plays an important role in the pathogenesis of melanoma. This pathway is physiologically activated when extracellular signals bind to their cognate membrane receptor, typically a receptor tyrosine kinase (RTK). Subsequently activated tyrosine receptor kinase leads to stimulation of small cytoplasmic proteins of RAS gene family (HRAS, NRAS and KRAS). Among them, NRAS mutations are found in about 10-15% of melanoma patients. Activated RAS results in a cascade of phosphorylation events involving the serine/threonine kinases RAF (encoded by ARAF, BRAF and CRAF). Activated RAF kinases phosphorylate and activate MEK1/2, which in turn phosphorylate and activate ERK1/2, leading to cellular proliferation, survival, and differentiation, and to an inhibitory feedback toward upstream components of the pathway (Fig. 1). About 50% of melanomas harbor an activating mutation in BRAF, the most common being BRAF V600E, which renders the kinase constitutively active [40-42].

## Vemurafenib

Vemurafenib is a potent inhibitor of the kinase domain in mutant BRAF, a mutation carried by half of melanomas. It was approved by US FDA and European Medicines Agency for the treatment of unresectable or MM with mutant BRAF V600E at a dose of 960 mg orally twice a day.

### Efficacy

A multicenter, phase I dose-escalation trial of 55 patients (49 with melanoma) established a phase II recommended dose of 960 mg twice daily [43]. In an extension phase of this trial, 32 patients with previously treated BRAF V600E-mutant MM demonstrated a tumor response rate of 81% (n = 26), with two patients demonstrating a CR. Among patients with symptomatic disease, improvement in symptoms was reported within 1 - 2 weeks.

A follow-up, multicenter, phase II study enrolled 132 patients with previously treated BRAF V600-mutant MM without brain metastases [44]. Patients who received vemurafenib 960 mg twice daily demonstrated an ORR of 53% (95% CI: 44-62%), with an additional 29% of patients achieving some degree of tumor control. The median PFS was 6.8 months and the median OS was 15.9 months.

In the international, multicenter, randomized, phase III BRIM-3 trial, 675 patients with previously untreated, MM with the BRAF V600E mutation were randomly assigned to either vemurafenib (960 mg twice a day) or dacarbazine (1,000 mg/m^2^ IV Q3W) in patients who had either metastatic disease or unresectable stage IIIC disease. The results of this study showed that OS was significantly prolonged with vemurafenib compared with dacarbazine (13.6 versus 9.7 months). PFS was also significantly prolonged (6.9 versus 1.6 months) [45]. Vemurafenib produced improved rates of OS and PFS in patients with previously untreated melanoma with the BRAF V600E mutation.

### Development of resistance

Several mechanisms have been hypothesized regarding the development of resistance to vemurafenib after a certain period of treatment. Mechanisms of primary resistance include RAC1P29S mutations (RAC 1 regulates cell proliferation and migration), COT overexpression (COT activates ERK through mechanism that does not depend on RAF signaling), alterations in RTK signaling (RTK activation can signal either through CRAF or through the PI3K pathway), and alterations in the PI3K-AKT-mTOR pathway (loss of function of PTEN). The reactivation of the MAPK pathway is the most frequent cause of acquired/secondary resistance; it may be driven by events that occur upstream (upregulation and activation of the RTK, NRAS activating mutations) or downstream (activating MEK1/2 mutation, or at the level of BRAF) [46].

Another mechanism that has been proposed is the overexpression of eIF4E which is a translation initiation factor. eIF4E is overexpressed in a panel of melanoma cell lines, compared to immortalized melanocytes. Knock-down of eIF4E significantly repressed the proliferation of a subset of melanoma cell lines. Moreover, in BRAF V600E melanoma cell lines, vemurafenib inhibits 4E-BP1 phosphorylation, thus promoting its binding to eIF4E. Cap-binding and polysome profiling analysis confirmed that vemurafenib stabilizes the eIF4E-4E-BP1 association and blocks mRNA translation, respectively. Conversely, in cells with acquired resistance to vemurafenib, there is an increased dependence on eIF4E for survival, 4E-BP1 is highly phosphorylated and thus eIF4E - 4E-BP1 associations are impeded. Moreover, increasing eIF4E activity by silencing of 4E-BP1/2 renders vemurafenib responsive cells more resistant to BRAF inhibition [47].

### AEs

The most common TEAEs were arthralgia, rash, nausea, photosensitivity, fatigue, cutaneous squamous cell carcinoma, pruritus and palmar-plantar dysesthesia in the phase I trial [43]. Increased incidence of squamous cell carcinoma in these patients resulted from increased proliferation of HRAS-mutant cell lines exposed to vemurafenib which was associated with a paradoxical re-activation of MAPK signaling [48]. A vemurafenib analog (PLX4720) accelerated the growth of the lesions harboring HRAS mutations, and this growth was inhibited by concomitant treatment with an MEK inhibitor. The common AEs in phase II and phase III trials were cutaneous events, arthralgia, rash, fatigue, alopecia, photosensitivity, nausea and diarrhea [44, 45].

A few case reports have been published in the literature where vemurafenib is associated with development of uveitic cystoid macular edema [49], Fanconi syndrome [50], gingival hyperplasia [51], peripheral facial palsy [52], severe radiation dermatitis [53] and prolongation of QT interval.

## Dabrafenib

Dabrafenib is another BRAF kinase inhibitor that has demonstrated significant activity in patients with advanced melanoma compared with dacarbazine chemotherapy. Dabrafenib was approved by the US FDA in May 2013 for the treatment of patients with advanced melanoma that contains the V600E mutation of BRAF at a dose of 150 mg orally twice a day.

### Efficacy

An initial phase I trial of patients with BRAF V600E-mutant melanoma examined escalating doses of dabrafenib in 184 patients (156 patients with MM and 28 with non-melanoma solid tumors) [54, 55]. This trial established a dose of 150 mg orally twice daily for further studies.

A phase II study (BREAK-2) evaluated the use of dabrafenib in patients with BRAF-mutant treatment-naive MM. A total of 76 patients with BRAF V600E and 16 patients with V600K mutations were treated with dabrafenib 150 mg twice daily. BRAF V600E patients had a confirmed response rate of 59% (95% CI: 48-70%) with a 7% CR rate (n = 5), whereas patients with the BRAF V600K mutation had a response rate of 13% (95% CI: 0-28%). Median OS was 13.1 months for the V600E patients and 12.9 months for the V600K patients [56].

An open-label, multicenter phase II trial was conducted in patients with brain metastases to assess the efficacy of dabrafenib (BREAK-MB) [57]. One hundred seventy-two patients were enrolled and split into two cohorts; those with no prior local treatment for brain metastases (cohort A, n = 89) and those who had progressed after previous local treatment (cohort B, n = 83). Patients were treated with 150 mg of dabrafenib twice daily. An intracranial response was observed in 39% (95% CI: 28-51%) in cohort A and 30% (95% CI: 20-43%) in cohort B patients.

In the pivotal phase III trial, 250 previously untreated patients with unresectable stage III or stage IV melanoma were randomly assigned in a 3:1 ratio to either dabrafenib (150 mg orally twice a day) or dacarbazine (1,000 mg/m^2^ IV Q3W). All patients had the V600E mutation in BRAF, the patients in the dacarbazine arm being allowed to crossover at the time of disease progression. Dabrafenib significantly improved median PFS to 5.1 months as compared to 2.7 months for dacarbazine (hazard ratio (HR) = 0.30, P < 0.001). The confirmed response rate was 50% for dabrafenib, with a 3% (n = 6) CR rate, and a median time to response of 6.3 weeks [58]. Dabrafenib significantly improved PFS compared with dacarbazine.

### AEs

The most common grade 2 or higher AEs in the phase I trial were cutaneous squamous cell carcinoma or keratoncanthoma, fatigue and pyrexia [54]. Common toxicities in the phase II trial (BREAK-2) included arthralgia, hyperkeratosis, pyrexia, fatigue and headache. Serious AEs were reported in 25 patients (27%) and these included basal cell carcinoma, cutaneous squamous cell carcinoma, anemia, pyrexia, non-cardiac chest pain and vomiting [56]. The common AEs in phase III trial were cutaneous events, rash, fatigue, headache and arthralgia [58]. Dose reduction was required in 28% (n = 52) of patients, and five patients (3%) discontinued the drug.

### Usage in other cancers

Dabrafenib is also used in the treatment of hairy cell leukemia along with melanoma [59]. Dabrafenib is used in the treatment of melanoma in patients with leucopenia induced by vemurafenib as dabrafenib has no negative influence on leucocyte count [60]. Dabrafenib can be used as a substitute for vemurafenib if the patient develops severe cutaneous AE like toxic epidermal necrolysis from vemurafenib requiring its discontinuation [61]. Dabrafenib was well tolerated and resulted in durable responses in BRAF-mutant differentiated thyroid carcinoma patients [62].

## Trametinib

Trametinib, an MEK inhibitor, was first approved by FDA in 2013 for treatment of patients with unresectable or MM with BRAF V600E or V600K mutation. Trametinib is usually taken as 2 mg orally once daily as a single agent or 2 mg taken orally once daily with dabrafenib 150 mg orally taken twice.

### Efficacy

Based on the below mentioned trial, trametinib was approved by the FDA in 2013 for the treatment of patients with unresectable stage IIIC or MM with BRAF V600E/K mutations.

In an open-label phase III trial (METRIC), 322 patients with BRAF V600E/K-mutant MM were randomized to receive either trametinib (n = 214) or chemotherapy with either dacarbazine or paclitaxel (n = 108) [63]. Patients who received prior BRAF inhibitors or MEK inhibitors were excluded from study. Patients in the chemotherapy group were allowed to crossover to trametinib with disease progression. The primary endpoint is median PFS which was improved to 4.8 months in the trametinib group, as compared to 1.4 months in the chemotherapy group (HR = 0.45, 95% CI: 0.33 - 0.63; P < 0.001). The OS rate at 6 months was 81% in the trametinib group and 67% in the chemotherapy group (HR = 0.54, 95% CI: 0.32 - 0.92; P = 0.01), despite 65% of patients in the chemotherapy arm crossing over to the trametinib arm.

Prior to this trial, there was a phase I trial looking at pharmacodynamics, dose escalation and AEs.

The phase I trial study was done in three parts: firstly, dose escalation to define the maximum tolerated dose (MTD) followed by identification of the recommended phase II dose, and lastly assessment of pharmacodynamic changes. The study included 206 patients who had any solid tumors, of which 97 patients were diagnosed with MM. Blood samples and tumor biopsy specimens were taken to assess pharmacokinetic and pharmacodynamic changes. AEs were defined with common toxicity criteria, and tumor response was measured by response evaluation criteria in solid tumors (RECIST). Overall ORR was 10%. However, BRAF-mutant melanoma had a response rate of 33% [64]. The RECIST-defined response rate was 40% in the 30 patients with BRAF inhibitor-naive MM, but only 17% in those with prior BRAF inhibitor therapy (n = 6). This study has recommended a dose of 2 mg once a day with manageable side effects.

### AEs and safety

The most common AEs with trametinib are skin-related toxicities followed by diarrhea and less common included ocular toxicities and decrease in left ventricular ejection fraction. These are explained in detail below.

In the phase I dose-escalation study of trametinib, a loading dose regimen of two 10 mg/day loading doses followed by 3 mg/day led to grade 3 diarrhea, grade 3 rash and grade 2 central serous retinopathy. Of the 70 patients treated with the phase II recommended dose of 2 mg/day, only eight (11%) had grade 3 treatment-related events requiring dose reduction. The most typical event leading to dose reduction was rash, which was poorly classified but most often was acneiform [54, 64]. Although skin-related toxicities were common, no events of cutaneous squamous cell carcinoma or other proliferative skin lesions were recorded. Diarrhea was predominately grade 1 and was manageable with standard symptom-based therapies. Treatment-related ocular toxicities were recorded in 31 patients (15%), including one episode of retinal vein occlusion at the phase II recommended dose of 2 mg/day. The visual acuity of this patient improved after intraocular treatments of antibodies against VEGF. A decline in left ventricular ejection fraction was noted in 16 (8%) patients, but most of these events were grade 2 or lower. The mechanism of reduced left ventricular ejection fraction in relation to MEK inhibition is unknown, but cardiac toxicity has previously been reported in association with MEK inhibitors; this could represent a class effect [65]. Subject’s cardiac function did return to baseline after discontinuation of trametinib.

In the phase III trial, the common AEs associated with trametinib therapy were rash, diarrhea, peripheral edema, fatigue and dermatitis [63]. A decrease in left ventricular ejection fraction was noted in 14 patients (7%) and two patients (1%) had serious cardiac-related events that were considered to be drug-related, prompting drug discontinuation. Ocular events (mostly grade 1 or 2) occurred in 9% of patients, with no observed cases of retinal vein occlusion. In total, AEs led to treatment interruption in 35% of patients and to dose reduction in 27%.

## Combination Therapy

MAPK pathway inhibition by either BRAF or MEK inhibitors has proven to increase PFS and OS in patients with MM. Their efficacy has been curtailed by the toxicity of the side effects and development of resistance in patients as well as the emergence of secondary cancers such as cutaneous squamous cell carcinoma due to the activation of BRAF inhibitor-induced paradoxical activation of MAPK pathway. Combination therapy with BRAF and MEK inhibitors is displaying potential to overcome these roadblocks. The studies below demonstrate that the combination therapies decrease the advent of secondary cancers, while delaying the emergence of resistance and increasing the PFS and OS compared to monotherapy [66, 67].

## Combined BRAF and MEK Inhibitor

### Dabrafenib and trametinib versus dabrafenib and placebo

In a phase III double-blinded study conducted by Long et al [68], 423 patients with unresectable stage IIIC or stage IV melanoma with BRAF V600E/K mutation were randomized to receive the combination of oral BRAF inhibitor dabrafenib and MEK inhibitor trametinib (n = 211) (combination therapy group) or dabrafenib and placebo (n = 212) (dabrafenib group). Patients who had previous systemic anticancer treatment and those without the BRAF V600E/K mutation were excluded from the study. The patients were stratified according to the BRAF genotype and baseline LDH level. The primary endpoint was PFS and the secondary endpoints were OS, response rate, response duration, safety and pharmacokinetics.

#### Efficacy

In the intention-to-treat group, the estimated median PFS was longer in the combination therapy group compared to the dabrafenib only group (9.3 vs. 8.8 months with HR of 0.75, P = 0.03). In the patient population with elevated LDH levels, the median PFS was longer in the combination therapy group compared to monotherapy (7.1 vs. 3.8 months). The ORR was 67% in the combination therapy group versus 51% in the monotherapy group. Ten percent of patients achieved CR and 56% had PR in the dabrafenib-trametinib group compared to 9% of patients with CR and 43% with PR in the dabrafenib only group.

Quality of life and pain management are important factors in patients with non-curative cancers such as MM. The study done by Schadendorf et al showed that the combination therapy is shown to have superior conservation of health-related quality of life and improvement in pain compared to monotherapy [69].

#### AEs

In both combination therapy and monotherapy groups, pyrexia, fatigue, headache, nausea, diarrhea and arthralgia were the most commonly reported AEs. However, pyrexia, hypertension, peripheral edema and diarrhea were more common in the combination group than the monotherapy group. On the other hand, the incidence of alopecia, papillomas and hand and foot syndrome was lower in the dabrafenib-trametinib group versus dabrafenib alone. Secondary cancers such as cutaneous squamous cell carcinoma were also lower in the combination therapy group vs. monotherapy (2% vs. 9%).

### Vemurafenib and cobimetinib versus vemurafenib and placebo

A phase III randomized trial conducted by Larkin et al [70] on 495 patients with BRAF V600E/K-mutated metastatic or locally invasive melanoma showed similar results to the above study. Patients who had received prior cancer treatment and those with wild type BRAF mutation were excluded from the study. Patients were randomly assigned to receive either combination therapy of oral BRAF inhibitor vemurafenib (960 mg twice daily) along with oral MEK inhibitor cobimetinib (60 mg once daily) or monotherapy of oral vemurafenib along with placebo. The primary endpoint was PFS and the secondary endpoints were OS, response rate, response duration and safety. The results reported are from July 2014.

#### Efficacy

The combination group had a significantly higher PFS compared to the control group (9.9 vs. 6.2 months, 95% CI: 5.6 - 7.4). The HR was determined to be 0.51 (95% CI: 0.39 - 0.68; P < 0.001). The OS at 9 months for the combination group was 81% compared with 73% of the control group. The response rate was determined to be higher in the combination group compared to the control group 68% vs. 45% (P < 0.001). CR was also higher in the combination group vs. control group (10% vs. 4%) and the median response duration was 7.3 months in the combination group while the control group did not reach the median.

#### AEs

Central serous retinopathy, elevated aminotransferase, creatinine kinase levels and gastrointestinal symptoms occurred at a higher rate in the combination group compared to the control group. The majority of these events were either grade 1 or 2.

### Ipilimumab plus sargramostim vs. ipilimumab

Ipilimumab is an IgG1 monoclonal antibody that blocks CLTA-4 thereby enhancing T-lymphocyte activity. Sargramostim is a granulocyte monocyte colonoy stimulating factor (GM-CSF) cytokine that augments antigen presentation by dendritic cells thereby improving lymphocyte antitumor activity [11]. In a phase II randomized clinical trial study by Hodi et al [71], 245 patients with stage III or IV melanoma with at least one prior therapy were randomized to receive CTLA-4 blockade with ipilimumab and GM-CSF secreting tumor vaccine sargramostim (n = 123) or ipilimumab (n = 122) alone. The primary objective was OS and the secondary endpoints were PFS, response rate, safety and the tolerability. Median time of follow-up was 13.3 months. The OS data as of December 2012 and other data as of March 2013 were reported.

#### Efficacy

The median OS for the combination group was 17.5 months (95% CI: 14.9-not reached) versus 12.7 months (95% CI: 10.0-not reached) for the monotherapy group. The 1-year survival rate for the ipilimumab-sargramostim group was 68.9% (95% CI: 60.6-85.5%) compared to 52.9% (95% CI: 43.6-62.2%) for the ipilimumab only group. Subgroup analysis showed that men who were treated with ipilimumab plus sargramostim had greater OS compared to ipilimumab alone while the opposite trend was seen in women. Caution should be used when interpreting the subgroup results as the sample size and number of deaths in subgroups was comparatively small. However in PFS and response rate, there was no statistical difference between the two groups.

#### AEs

Grade 3-5 events occurred in 44.9% of ipilimumab-sargramostim group compared to 58.3% in the ipilimumab group. Gastrointestinal toxicities such as colonic perforation and pulmonary toxicity were notably decreased in the ipilimumab-sargramostim group versus the ipilimumab group (16.1% vs. 26.7% and 0% vs.7.5%) respectively.

### Other combinations

#### Vemurafenib and ipilimumab

In a phase I study conducted by Ribas et al [72], the first cohort of six patients each received full dose of vemurafenib (960 mg twice daily) for 1 month followed by ipilimumab infusions Q3W along with twice daily dose vemurafenib. Four of the six patients developed dose-limiting, grade 3 increase in aminotransferase levels 2 - 5 weeks after first infusion of ipilimumab.

A second cohort of six patients received decreased dose of vemurafenib (720 mg twice daily) with full dose of ipilimumab. Two of the patients developed grade 3 elevations and one developed grade 2 elevation of aminotransferase 3 weeks after ipilimumab infusion. The study was discontinued secondary to hepatotoxicity of the combination therapy.

#### Nivolumab plus ipilimumab

In a phase I trial conducted by Wolchok et al [73], 53 patients received anti-CTLA-4 antibody ipilimumab and anti-PD-1 receptor antibody nivolumab concurrently while 33 patients received sequenced treatment. All patients had stage III or IV unresectable melanoma. Ninety-three percent of patients in concurrent group had TEAEs compared to 73% in the sequenced treatment group. Most common AEs were rash, pruritus, fatigue and diarrhea. Dose-limiting grade 3 or 4 events were noted in 21% of patients in the concurrent group vs. 9% in the sequenced group. Most drug-related events were treated with immunosuppressant. The data from this study showed that the combination therapy had an acceptable safety profile and warrants further investigation to compare efficacy of the combination therapy versus monotherapy in advanced melanoma.

Postow et al [74] recently conducted a double-blinded study involving 142 patients (109 with BRAF wild type and 33 with BRAF V600E mutation positive) with MM who had not previously received treatment. They were randomly assigned in a 2:1 ratio to receive ipilimumab (3 mg/kg body weight) combined with either nivolumab (1 mg/kg) or placebo once Q3W for four doses, followed by nivolumab (3 mg/kg) or placebo Q2W until the occurrence of disease progression or unacceptable toxic effects. The primary endpoint was the rate of investigator-assessed, confirmed objective response among patients with BRAF V600 wild-type tumors.

Among patients with BRAF wild-type tumors, the rate of confirmed objective response was 61% (44 of 72 patients) in the group that received both ipilimumab and nivolumab (combination group) versus 11% (four of 37 patients) in the group that received ipilimumab and placebo (ipilimumab-monotherapy group) (P < 0.001), with CR reported in 16 patients (22%) in the combination group and no patients in the ipilimumab-monotherapy group. The median duration of response was not reached in either group. The median PFS was not reached with the combination therapy and was 4.4 months with ipilimumab monotherapy. Similar results for response rate and PFS were observed in 33 patients with BRAF mutation-positive tumors. Drug-related AEs of grade 3 or 4 were reported in 54% of the patients who received the combination therapy as compared with 24% of the patients who received ipilimumab monotherapy. The ORR and the PFS among patients with advanced melanoma who had not previously received treatment were significantly greater with nivolumab combined with ipilimumab than with ipilimumab monotherapy.

Recently Larkin et al [75] conducted a double-blinded, phase III trial comparing nivolumab alone or nivolumab plus ipilimumab to ipilimumab alone in untreated patients with advanced MM. Nine hundred forty-five patients were randomly assigned in a 1:1:1 ratio to either nivolumab alone or nivolumab plus ipilimumab or ipilimumab alone. The primary endpoints were PFS and OS. The median PFS was 11.5 months in patients with nivolumab plus ipilimumab, 2.9 months in ipilimumab alone (P < 0.001) and 6.9 months in nivolumab alone (P < 0.001). In patients with tumors positive for PD-L1, the median PFS was 14.0 months in both nivolumab plus ipilimumab and nivolumab alone groups, whereas in patients with PD-L1 negative tumors, PFS was longer with combination therapy than nivolumab alone. TEAEs of grade 3 or 4 occurred in 16.3% of the patients in the nivolumab group, 55.0% of those in the nivolumab-plus-ipilimumab group, and 27.3% of those in the ipilimumab group.

This study proved that in previously untreated patients with MM, nivolumab alone or nivolumab with ipilimumab resulted in significantly longer PFS than ipilimumab alone. In patients with PD-L1-negative tumors, the combination of PD-1 and CTLA-4 blockade was more effective than either agent alone.

## Conclusion and Approach to Therapy

In summary, the survival rate of patients with MM far improved by the development of these novel drugs and drug combinations. Anti-PD-1 antibodies like pembrolizumab and nivolumab have become the preferred approach to immunotherapy in patients with advanced melanoma, even though they are associated with various autoimmune AEs. Anti-CTLA-4 antibody like ipilimumab retains a role in combination with anti-PD-1 antibodies. Targeted therapy against MAPK pathway is an important option for the treatment of patients with characteristic BRAF V600 mutation. Targeted therapy is not indicated in patients without a characteristic V600 mutation.

The choice to use either immunotherapy or targeted therapy or combination therapy depends on the performance status of the patient and BRAF V600 mutation.

For patient with a BRAF V600 mutation and good performance status, immunotherapy is recommended initially rather than targeted therapy. Immunotherapy in the form of nivolumab and ipilimumab combination is recommended as the initial systemic therapy. For those patients whose disease can no longer be controlled with this immunotherapy, targeted therapy using a combination of BRAF inhibitor/MEK inhibitor (dabrafenib/trametinib) is recommended.

For patients with BRAF V600 mutation and bulky disease, elevated serum LDH, visceral metastases, poor performance status, targeted therapy is initially recommended. Immunotherapy is also an alternative and may be recommended after progression on targeted therapy in those patients.

For patients without BRAF V600 mutation and good performance status, immunotherapy that includes combination of nivolumab and ipilimumab is initially recommended. Anti-PD-1 monotherapy with either nivolumab or pembrolizumab is a suitable alternative when toxicity is a concern with combination therapy. For poor performance status patients without BRAF V600 mutation, who are not thought to be able to tolerate combination treatment, single agent anti-PD-1 therapy is recommended.

The advent of immune and targeted therapy has ushered in an era of optimism for the treatment of MM. Monotherapy regimens with these new pharmaceuticals are hampered by the emergence of dose-limiting toxicity and secondary cancers. Although some of these concerns are addressed by the combination therapies, further studies are needed to evaluate triple combination therapies and new immune modulators and targeted therapy.

In summary, the treatment of advanced-staged melanoma is a rapidly developing field. Several new and effective treatments have been introduced in recent years, to the benefit of melanoma patients of all ages. With an increasing incidence of melanoma, particularly among older patients, their participation in clinical trials is essential.
